# Detecting suicidality among adolescent outpatients: evaluation of trained clinicians' suicidality assessment against a structured diagnostic assessment made by trained raters

**DOI:** 10.1186/1471-244X-8-97

**Published:** 2008-12-31

**Authors:** Matti Mikael Holi, Mirjami Pelkonen, Linnea Karlsson, Virpi Tuisku, Olli Kiviruusu, Titta Ruuttu, Mauri Marttunen

**Affiliations:** 1 Department of Mental Health and Alcohol Research, National Public Health Institute, Helsinki, Finland; 2 Kellokoski Hospital, Kellokoski, Finland; 3 Department of Psychiatry, Peijas Hospital, Helsinki University Central Hospital, Helsinki, Finland; 4 Department of Psychiatry, University of Kuopio, Kuopio, Finland

## Abstract

**Background:**

Accurate assessment of suicidality is of major importance. We aimed to evaluate trained clinicians' ability to assess suicidality against a structured assessment made by trained raters.

**Method:**

Treating clinicians classified 218 adolescent psychiatric outpatients suffering from a depressive mood disorder into three classes: 1-no suicidal ideation, 2-suicidal ideation, no suicidal acts, 3-suicidal or self-harming acts. This classification was compared with a classification with identical content derived from the Kiddie Schedule for Affective Disorders and Schizophrenia (K-SADS-PL) made by trained raters. The convergence was assessed by kappa- and weighted kappa tests.

**Results:**

The clinicians' classification to class 1 (no suicidal ideation) was 85%, class 2 (suicidal ideation) 50%, and class 3 (suicidal acts) 10% concurrent with the K-SADS evaluation (γ^2 ^= 37.1, df 4, p = 0.000). Weighted kappa for the agreement of the measures was 0.335 (CI = 0.198–0.471, p < 0.0001). The clinicians under-detected suicidal and self-harm acts, but over-detected suicidal ideation.

**Conclusion:**

There was only a modest agreement between the trained clinicians' suicidality evaluation and the K-SADS evaluation, especially concerning suicidal or self-harming acts. We suggest a wider use of structured scales in clinical and research settings to improve reliable detection of adolescents with suicidality.

## Background

Suicide is a major cause of mortality among adolescents; it has been estimated that up to 25% of young people have had suicidal ideation, and approximately 2–12% have attempted suicide at some time in their lives [1]. Standardized clinical assessments of adolescent outpatient samples have revealed that up to 60% have suicidal ideation, and up to 20% have made suicide attempts [2,3]. In a sample of adolescent depressed outpatients more than half had made suicide attempts [4]. According to a review by Safer [5] anonymous surveys of suicidal behavior have yielded lifetime prevalences of 7% to 10% for adolescents, whereas studies using structured interviews have found lifetime prevalences of 3% to 4%. Regardless of the great variability in the estimations of its prevalence, suicidality in its different forms seems surprisingly prevalent in the adolescent general population. Adolescent suicide occurs mostly in the context of an active, often treatable, but unrecognized or untreated mental illness, such as depression or substance abuse [6,7]. The increase in antidepressant treatments of adolescents [8] have been suggested to at least partly explain the decline in the incidence of suicide [9] in many Western countries during the past decade. Recently, though, some reports have connected SSRI-treatment in adolescents to an increase in suicidality [10,11].

Suicide attempts are complex acts for which no single set of clinical characteristics can be expected to be a good predictor [12,13]. Although the domain of suicidal behavior is multidimensional [14], a continuum from suicide ideation to suicide attempts has been reported in clinical adolescent populations [15,3,16]. Although only a minority of patients with suicidal ideation attempt suicide, and only a minority of attempters die, a previous suicide attempt has been shown to be one of the most significant risk factors for suicide [17-19]. Research concerning the role of suicidal ideation as a risk factor for suicide is less consistent, but many studies suggest that suicidal ideation predicts suicide attempts and suicides [e.g. [2,20,21]]. Thus, accurate assessment of suicidality is of major importance in both clinical and research settings.

The ability of clinicians to evaluate suicidality has been addressed in a few publications. Pelkonen et al. [22], for example, found that previous and current suicidal behavior was more common than referring persons were able to recognize, and could be detected by the clinician's systematic, structured, and documented inquiring about suicidality of all adolescent psychiatric outpatients. In a study by Malone et al. [23] fewer suicide attempts were clinically reported than in concurrently and independently completed research data. These studies suggest that a significant degree of past and present suicidal behavior is not recognized and recorded during routine clinical assessment. Thus, easy-to-use instruments are needed to improve the clinicians' ability to recognize suicidality.

Numerous instruments have been developed with the aim of measuring different factors involved in the complex clinical task of suicide risk evaluation, but the use of them is often restricted to research settings [14]. A three-class mutually exclusive grouping of suicidality (non-suicidal, suicide ideation, suicide attempts) assessed by a clinician is a simplified version of the 5-item "Spectrum of Suicidal Behavior Scale" [24,25], and has previously been used in both research and clinical settings [3]. It consists of two structured questions, which are asked during a routine clinical interview, and the documentation of the answers to them. There is some evidence supporting the predictive validity of this grouping [3] but there have never been attempts to compare it with more structured measures.

We aimed to evaluate this simple and straightforward assessment of suicidality by training clinicians to ask about suicidality and document the answers, and compare this data with data obtained from the suicidality items of the K-SADS-PL Screen Interview. Although we expected these measures to converge with each other, we hypothesized that the clinical evaluation might under-detect suicidality compared with the structured assessment performed by trained raters.

## Methods

### Subjects

The subjects were 218 adolescent psychiatric outpatients. They suffered from depressive mood disorder, were of ages 13 – 19, and took part in the Adolescent Depression Study (ADS [26,27] karlsson et al 2008). They were recruited between March 1^st^, 1998 and December 31^st^, 2001 from a consecutive sample of patients attending the outpatient clinics of the Department of Adolescent Psychiatry of Peijas Medical Health Care District (PMCD) covering approximately 210,000 inhabitants and comprising the cities of Vantaa and Kerava in the Helsinki metropolitan area, Southern Finland. Of the eligible (appropriate age, knowledge of Finnish language, and adequate cognitive capacity) 660 outpatients, 624 (94.5%) were screened by the Beck Depression Inventory (BDI) [28] and the General Health Questionnaire-36 (GHQ-36) [29,30] during their first consultation visit. 373 patients (59.8%) with scores of 10 or more and 5 or more, respectively, were considered screen positive, and were asked to participate in the study. 118 (31.6%) outpatients refused to participate and 34 (9.1%) dropped out at this stage. 221 (33.5%) remaining outpatients were evaluated by a diagnostic interview (K-SADS-PL) [31]; 218 (33.0%) with a current depressive mood disorder were included in the study. A written informed consent was obtained from the subjects. For subjects less than 18 years old consent was asked from parents or other legal guardians. The study protocol was accepted by the ethics committees of Helsinki University Central Hospital and PMCD.

### Measurements

1. Clinicians' suicidality assessment (CSA), a three-point mutually exclusive grouping of suicidality (1-non-suicidal, 2-suicide ideation, 3-suicide attempts) is a simplified version of the 5-item "Spectrum of Suicidal Behavior Scale" [24,25]. It has been used for both research and clinical purposes [3]. The grouping is done by a clinician, and is based on two simple questions "Have you thought of killing yourself?" and "Have you attempted suicide?" and on patient records when appropriate. In this study, after a brief training, the treating clinicians of the outpatient clinics made the clinical suicidality assessment. They were instructed to include in class 3 also self-mutilation and other self-harming behavior whether or not actual suicide intent was evident.

2. K-SADS-PL: The Kiddie Schedule for Affective Disorders and Schizophrenia for School-Aged Children-Present and Lifetime (K-SADS-PL) [31] is a widely used semi-structured diagnostic interview with fairly good psychometric properties [31,32]. It is considered an internationally reliable and valid diagnostic instrument for adolescent population [33]. K-SADS-PL has been translated into Finnish and then back translated to confirm accuracy of translation. It has been used in numerous studies on adolescents in Finland [e.g. [34]]. In this study the five items concerning suicidal ideation and behavior of the Screen Interview of the K-SADS-PL were used as the standard for assessing suicidality. The items inquire about thoughts of death (item 1), suicidal ideation (item 2), presence of suicide attempts (item 3), non-suicidal self-harming behavior (item 4), and medical lethality and intent associated with the possible suicide attempt (item 5). Each item is scored using a 0- to 3-point rating scale. Score of 0 indicates that no information is available, a score of 1 suggests the symptom is not present, a score of 2 indicates sub-threshold level of the symptom, and a score of 3 indicates threshold criterion. Nine trained raters, who were also experienced clinicians, did the rating. Inter-rater reliability, assessed using 15 randomly selected videotaped interviews, was good for mood disorder diagnoses [weighted kappa [35] for MDD, other mood disorder, no mood disorder 0.87 (95% CI = 0.81–0.93)].

### Procedures

Both assessments were performed at the beginning of treatment in an outpatient clinic, and the time frame for suicidality for both instruments was the preceding 2 weeks. To enable a meaningful comparison between the two instruments, the results of the K-SADS-PL were forced into three classes identical to the above-mentioned clinical suicidality assessment as follows: Class 1 – no suicidal ideation in K-SADS-PL item 1 and 2; Class 2 – suicidal ideation in K-SADS-PL item 1 or 2, but no suicidal or self-harming acts according to K-SADS-PL items 3–5; Class 3 – suicidal or self harming acts according to K-SADS items 3–5, regardless of the score in item 1 and 2. Only scores of 3 in each item indicating presence of threshold criterion were counted. This procedure ensured that the three-class classification with increasing severity of suicidality by the two instruments was practically identical in their content.

Two comparisons were made, with two different K-SADS-PL definitions of suicidal ideation: 1) with thoughts of death not included in suicidal ideation, and 2) with thoughts of death included in suicidal ideation.

The K-SADS-PL results of the cases that were classified inconsistently by the two measures were explored item by item. Lifetime suicidal or self-harming acts detected clinically (from the patient records) and by the K-SADS-PL were estimated and compared.

### Statistical analysis

The convergence in the classification by the two measures was calculated by Kappa (κ) and weighted kappa analysis [35]. The sensitivity and the specificity of the CSA to recognize suicidality were assessed against the K-SADS-PL. Statistical analyses were performed by the SPSS 11.0 [36]. Calculations of weighted kappa were performed by the SAS [37].

## Results

### Descriptive data

All the subjects were classified by both the CSA and the K-SADS-PL. The subjects' mean age was 16.4 (SD 1.6), 18% (n = 40) were boys and 82% (n = 178) girls. The median interval between the two measurements was 6 days (range 0–35). The interval did not influence the correlation between the measurements (r = .369, p = .000 vs. r = .364, p = .000). Thirty (13.8%) subjects were classified by the K-SADS-PL as having had suicidal or self-harming acts (Class-3) during the past two weeks, 32 (14.7%) as having suicidal ideation (Class-2), and 156 (71.6%) as having no suicidality (Class-1). After including recurrent thoughts of death, suicidal ideation was present in 52 (23.9%) subjects.

### Convergence of the measurements

Seventy percent (n = 152) of the subjects were classified identically by the two three-class suicidality assessments (χ^2 ^= 57.8, df 4, p = 0.000). Weighted kappa [35] for the agreement of the two classifications was 0.335 (95% CI = 0.198–0.471, p < 0.0001). Kappa on the agreement about any form of suicidality was 0.404 (p < 0.0001). In a comparison between CSA and the K-SADS-PL classification with thoughts of death included in suicidal ideation 67.4% (n = 147) of the subjects were classified identically (χ^2 ^= 44.5, df 4, p = 0.000, weighted κ = 0.346, 95% CI = 0.215–0.492, p < 0.0001). Comparisons of the classifications are presented in Table 1.

**Table 1:** Convergence of three-class clinical suicidality assessment (CSA) made by treating clinician and K-SADS-PL suicidality scoring performed by trained rater in 218 adolescent outpatients with a mood disorder.

		**K-SADS-PL**		
	**No suicidality**	**Suicidal ideation**	**Suicidal acts**	TOTAL
**CSA – no suicidality**	133 (121)	15 (27)	15	163
**CSA – suicidal ideation**	22 (14)	16 (24)	12	50
**CSA-suicidal acts**	1 (1)	1 (1)	3	5
TOTAL	156 (136)	32 (52)	27	218

(comparison with K-SADS-PL detected thoughts of death included in suicidal ideation is in parentheses).

### Validity coefficients

The sensitivity and specificity of CSA in recognizing any suicidality (thoughts or acts) were 51.6% and 85.3%, respectively, using K-SADS-PL (without thoughts of death in suicidal ideation) as the standard. The sensitivity and specificity of CSA in recognizing suicidal or self-harming acts were 10% and 98.9%, respectively. Twenty-one (77.8%) of the 27 K-SADS-PL class-3 cases missed by the CSA had non-suicidal self-harming and suicidal acts, as thirteen of them were reported having had only non-suicidal self-harming without suicidal acts.

### Lifetime suicidality

Seventy-eight (35.8%) subjects by the K-SADS-PL and 100 (45.9%) subjects by clinical records were classified as having had suicidal or self-harming behavior during their lifetime. The convergence between these estimates was significant (χ^2 ^= 143.3 df 1 p = 0.000, κ = 0.79) although 22 (10.1%) patients were classified incorrectly.

## Discussion

In order to improve the clinicians' ability to detect suicidality, we trained them to ask about suicidality and to document the answers. This study assessed the utility of this simple and straightforward 3-class clinical suicidality assessment against a semi-structured diagnostic interview (K-SADS-PL). We found that although the classifications converged significantly, the agreement between these two ways of evaluating suicidality, especially concerning suicidal or self-harming acts, was not satisfactory.

### Suicidal acts and self-harming

Over 3/4 of the suicidal subjects, undetected by the clinicians, had self-harming acts, about half of them having self-harm without suicidal acts. According to this study, deliberate self-harm without clear suicidal intention seems especially difficult for clinicians to detect using the crude "suicidal acts" classification. Furthermore, our results suggest that self-harm may even lead to under-detection of co-occurring suicidal acts. It is possible that self-harm may not be regarded as "true suicidality" (either by the patient or by the clinician), and it may "mask" suicidal acts from the clinician. Deliberate self-harm may be under-recognized by the treating personnel, unless it is specifically inquired [22], as adolescents tend not to inform others of it [38]. Adolescent patients may underreport suicidality, especially deliberate self-harm due to concerns about confidentiality [5]. Questionnaires or rating scales with a rater from outside the treatment team may be needed for appropriately sensitive detection of suicidal or self-harming acts.

### Lifetime suicide attempts

The K-SADS-PL, although detecting current suicidality, seems not to reliably detect lifetime suicidality, as it did not recognize 10% of subjects with suicidal behavior documented in patient records. This may be due to the fact that the suicidality items are inquired with questions concerning mood disorder episodes, and the most severe episode may not be the most suicidal if for example the patient has impulsive traits.

### Suicidal ideation

Different from self-harming, our results suggest that clinicians' evaluation over-detects suicidal ideation compared with the K-SADS-PL. Over 1/3 of the subjects over-detected by the clinicians had thoughts of death, and the rest had sub-threshold suicidal ideation. In clinical practice, accurate ruling out of thoughts of death with no thoughts of suicide may be difficult and may lead to over-detection of suicidal ideation. Clinicians may see thoughts of death as a form of suicidality that must be taken seriously. It may also be that thoughts of death could best be conceived as a mild form of suicidal ideation with a better prognosis than severe ideation [2]. Our results support this view, as after including thoughts of death in suicidal ideation of the K-SADS-PL there was a better convergence between the two instruments in detection of that form of suicidality. The overall convergence of the instruments did not improve, however, as the clinicians under-detected many subjects having thoughts of death.

The results suggest that K-SADS-PL diagnostic threshold for suicidal ideation may be too high; over half of the cases over-detected by the clinicians had sub-threshold suicidal ideation. K-SADS-PL classifies occasional thoughts of suicide without planning as sub-threshold suicidal ideation; frequent thoughts and planning are required to pass the threshold. Principally, sub-threshold suicidal ideation in the K-SADS-PL would qualify as suicidal ideation in the clinicians' evaluation that does not inquire about frequency. Thus, the over-detection of suicidal ideation by clinicians may be caused by the different thresholds and criteria of the two measures.

### Methodological concerns

1) The clinicians' evaluation here was based on two simple questions with good face validity, it was non-structured and naturalistic, and the classification is simple. However, there are some problems with this evaluation that deserve attention. First, in order to detect suicidal acts and self-harming more accurately a separate question for self-harming/self-mutilation with no suicidal intent may be needed. The assessment of intention of a suicide attempt is especially difficult in adolescents as they may not correctly perceive the lethality of their attempt [39]. Secondly, a more specific question or a higher threshold for the clinicians' inquiry about suicidal ideation may be needed, as they also seem to rate sub-threshold suicidal ideation as clinically significant.

2) The standard for suicidality detection in this study was the K-SADS-PL, which has a good face validity and satisfactory inter-rater reliability [32], but not enough overall psychometric support yet. Moreover, it has not yet been used widely in studies of suicidal behavior [40]. The K-SADS-PL was forced into three classes that suited the clinicians' evaluation well in their content. Expectedly, considering or not considering thoughts of death as suicidal ideation in the classification somewhat changed the convergence between the K-SADS-PL and the clinical rating. Our results suggest that the current diagnostic K-SADS-PL threshold for suicidal ideation may need adjustment downwards. According to our findings, the K-SADS-PL in its present form may underestimate lifetime suicidal acts or self-harming behavior. This may be due to its emphasis on episodes of mood disorders, which may not always be those with the most severe suicidality. In any case, studies on the reliability and validity of the assessment of suicidality using the K-SADS-PL are needed. As this study was cross-sectional it could not evaluate the crucial aspect of predictive validity in detecting suicidality.

3) Although large and representative, the sample was a pure outpatient sample, and females were over-represented. With inpatients included in the sample, we would probably have seen a somewhat wider spectrum of suicidality. As the sample was limited to an urban area in southern Finland, the generalizability of our findings to rural areas, or to other countries, is not known. Although the time frame for the two measurements was very similar, it was not identical, and this may cause some of the discrepancy. The screening performance of our measures may not be generalized into adult populations. Self-report questionnaires may be more sensitive in detecting suicidality in adolescent population because of confidentiality issues.

## Conclusion

This report compared clinical evaluation with structured evaluation in the detection of suicidality in a clinical population of adolescents, and brought valuable information concerning these different but commonly used ways of detecting suicidality. From the point of view of a clinician, it seems important that a measure of suicidality is sensitive in finding any suicidality. However, a good measure should detect also subjects with especially high risk in order to supply special interventions. The relation of thoughts of death and non-suicidal self-harming behavior to suicidality remains open and warrants further research. Studies on predictive validity would shed light on topics like the association of appropriate thresholds and different sub-threshold forms of suicidality to suicides and suicide attempts. Our results suggest that structured instruments are needed for appropriately sensitive detection of suicidal or self-harming acts and specific recognition of real suicidal ideation. We suggest that continuous training in inquiring about and detecting suicidality is needed in order to recognize subjects with suicidal acts and especially subjects with self-harming behavior. Furthermore, the development of simple and reliable measures of suicidality, which could easily be used by clinicians, is of major importance.

## Competing interests

The authors declare that they have no competing interests.

## Authors' contributions

MMH analyzed the data and wrote the paper. MP interviewed patients, participated in planning the study and analyses, and writing the paper. LK, TR and VT participated in planning the study, interviewed patients, and commented on the manuscript. OK participated in planning the study and the analyses, and commented on the manuscript. MM supervised the study, interviewed patients and participated in planning of the study and analyses, and writing the paper. All authors read and approved the final manuscript.

## Pre-publication history

The pre-publication history for this paper can be accessed here:


